# A prospective study comparing two embryo-transfer soft catheters

**DOI:** 10.5935/1518-0557.20170018

**Published:** 2017

**Authors:** Emily De Conto, Artur K Schuster, Vanessa K Genro, Rita Chapon, Daniela S da Silva, João Sabino Cunha-Filho

**Affiliations:** 1 Federal University of Rio Grande do Sul - UFRGS - Porto Alegre - Brazil; 2 INSEMINE Center of Human Reproduction, Porto Alegre/RS - Brazil

**Keywords:** Catheters, embryo transfer, pregnancy rate, assisted reproduction technology

## Abstract

**Objectives:**

To compare reproductive outcomes using two different soft catheters i.e. Set TDT^®^ and Cook^®^ Sydney IVF. The primary outcome was defined as a positive β-human chorionic gonadotropin (β-hCG) test.

**Methods:**

Our prospective study recruited 68 patients undergoing *in vitro* fertilization cycles in a private fertility clinic in Porto Alegre, Brazil, between January 2014 and April 2016. They were divided into two groups according to the catheter that would be used for the embryo transfer, and the groups were matched by age. The total number of patients in each group was: 34 for the TDT and 34 for the Cook Sydney. All the patients were submitted to a β-hCG test 12 days after the embryo transfer for pregnancy outcome evaluation.

**Results:**

Ten out of 34 patients from the TDT group had a positive outcome for pregnancy, corresponding to 29.4%. The Cook Sydney group had 9 patients out of 34 with positive outcomes, corresponding to 26.5%. Comparing the efficacy of both catheters for the primary outcome, there was no significant difference (*p*>0.05) between the TDT and the Cook Sydney catheters.

**Conclusion:**

The TDT and the Cook Sydney catheters efficacies were similar for embryo transfer during assisted reproductive technology cycles.

## INTRODUCTION

Embryo transfer (ET) is the last stage, and one of the most critical steps in Assisted Reproduction Technology (ART) cycles (^Sallam, 2005^). Among the most important variables that determine the success or failure of the procedure are: ultrasound guidance (^Brown *et al.*, 2016^), uterine contractions during ET (^Pierzyński & Zbucka-Kretowska, 2014^), and the catheter type used during the procedure (^Buckett, 2006^). A few years ago, the type of catheter chosen was recognized as one of the main variables interfering positively or negatively on the ET effectiveness. ^Kovacs (1999)^ and ^Salha et al. (2001)^ classify the selection of the catheter, respectively, and third and fourth most important variables for the positive outcome of the cycle.

Considering the importance of the catheter chosen, some studies have been carried out to compare the efficacy of those commercially available. The results of these studies showed that the soft catheters had better performance when compared to rigid or semi-rigid ones (^Abou-Setta *et al.*, 2005^; ^Ruhlmann *et al.*, 2015^), because they prevented trauma to the endocervix or the endometrium, and were pliable to be freely driven into the uterine cavity (^Omidi *et al.*, 2015^). When soft catheters were compared, the most used, and therefore the most studied, were the following: Edwards-Wallace (^McIlveen *et al.*, 2005^; ^Saldeen *et al.*, 2008^; ^Rhodes *et al.*, 2007^), Cook K-Jet (^McIlveen *et al.*, 2005^), Frydman Ultrasoft (^Ruhlmann *et al.*, 2015^) and Cook Sydney (^Saldeen *et al.*, 2008^).

The preferred catheter varies according to each professional's experience in embryo transfer. In our fertility clinic, two types of soft catheters are the most used: the Tight Difficult Transfer Set (Set TDT^®^) 4.5 and the Cook^®^ Sydney IVF catheters. We have used these catheters in most ET procedures and found that there were no studies comparing their effectiveness; thus, we decided to run a prospective study to compare two groups of patients undergoing embryo transfers using the TDT 4.5 and the Cook^®^ Sydney catheters.

## MATERIALS AND METHODS

### Patients

Sixty-eight women undergoing *in vitro* fertilization (IVF) cycles in our private fertility clinic (INSEMINE) in Porto Alegre, Brazil, were recruited for this prospective study. The recruitment period occurred between January 2014 and April 2016. The following criteria were considered for patient inclusion: regular ovulatory menstrual cycles every 25-35 days; both ovaries present; no clinical signs of hyperandrogenism; no current or past disease affecting ovaries or gonadotropin production and release, or sex steroid secretion, clearance or excretion; no current hormone therapy. Exclusion criteria involved: infertility caused by severe male factor and interrupted ART cycles. The age of the patients varied from 30 to 39 years. The most common cause of infertility among the patients was the male factor (38.2%, n = 26), followed by endometriosis (37.9%, n = 19), tubal factor (19.1%, n = 13) and other causes (14.8%, n = 10).

The patients were divided into two groups according to the catheter that would be used for their ET: Set TDT (group 1) and Cook Sydney IVT (group 2). The choice of catheter was made based on the operator's personal experience. Each group had a total of 34 patients.

This study was analyzed and approved by our institutional review board. All the patients signed consent forms and were made aware that their data could be used in trials that intended to improve AR.

### Hormonal Measurements and Laboratory Results

The primary outcome was defined as a positive β-human chorionic gonadotrophin (β-hCG) blood test. All the patients were submitted to this test 12 days after the ET. We considered as positive all the tests that had shown results above 25 mIU/mL. We also collected the following data: age, cause of infertility, antral follicular count (AFC), follicle-stimulating hormone (FSH), anti-Müllerian hormone (AMH), gonadotropin initial dose, number of follicles with diameter ≥ 17mm, retrieved oocytes, oocytes at metaphase II, fertilized oocytes in 2 PN, embryos generated, embryo score and number of embryos transferred (Table I).

**Table 1:** Comparison between two catheters for embryo transfer in relation to patient data and IVF cycle outcomes

	Catheters		*p* value
	Set TDT	Cook Sydney	
Number of AR cycles (n)	34	34	-
Age (y)	34.0±2.9	34.0±2.9	1.000
AFC	11.93±5.1	11.82±6.4	0.244
FSH (IU/L)	7.33±2.28	9.35±3.49	0.068
AMH (pmol/L)	3.99±3.54	4.15±4.05	0.921
Gonadotropin initial dose (IU)	258.6±51.4	234.4±67.5	0.040
Follicles with diameter≥17mm	4.88±3.7	5.19±3.5	0.988
Oocytes retrieved	8.00±4.6	7.69±3.8	0.199
Number at metaphase II	6.85±3.6	6.38±3.3	0.436
Fertilized oocytes in 2 PN	3.56±2.8	3.76±2.3	0.159
Generated embryos	4.21±2.7	4.88±2.9	0.823
Mean of embryo score	66.9±24.3	66.1±24.1	0.773
Number of embryos transferred	1.79±0.48	1.97±0.31	0.078
Pregnancy rate (%)	29.4	26.5	1.000

The results are presented as mean ± standard deviation. AFC: Antral Follicular Count, FSH: Follicle Stimulating Hormone, AMH: anti-Mullerian Hormone.

Each woman was submitted to blood sampling procedure by venipuncture on cycle day 3. The antagonist protocol was used for ovulation induction. We determined serum FSH levels using an automated multi-analysis system with chemiluminescence detection. Serum AMH levels were measured by an ultrasensitive enzyme-linked immunosorbent assay (ELISA). Laboratory data was obtained after oocyte puncture and embryo evaluation was performed according to ^Fisch *et al.* (2001)^ criteria.

### Catheters

Set TDT^®^ (Laboratoire CCD, France) is an extra thin soft transfer catheter. It has an outer sheath with a flexible extremity and 2 hysterometry guide-marks at 5.5 cm and 6.5 cm from the distal end. It also has a malleable metal stylet coated in polyethylene and an extra-thin transfer catheter on a stainless steel micro tube of 0.5 mm in diameter in its proximal section.

The Cook^®^ Sydney IVF (Cook Medical, USA) has an outer firm portion and an inner ultra soft portion. The outer catheter is 19 cm long and has a polycarbonate hub, a bulb tip and an angled distal end. The inner portion is 23 cm long and the tip is 2.8 French size.

### Embryo transfers

Embryo transfers were performed by a team of professionals formed by three gynecologists, all with over 5 years of experience. The difficult embryo transfers were excluded because they could influence the assisted reproduction cycle results.

### Statistical Analysis

We used the Statistical Package for Social Sciences (SPSS Inc., Chicago, IL, USA) Statistics 20.0 software for statistical analysis purposes. The statistical analysis for categorical variables was made using the Pearson Chi-Squared test. Continuous variables were analyzed by the Student's t test. The value considered as statistically significant was *p*≤.05.

## RESULTS

The study groups were matched by age and the following variables: AFC, FSH, AMH, number of follicles with diameter ≥ 17 mm, retrieved oocytes, oocytes in metaphase II, oocytes in 2 PN, generated embryos, mean score of embryos and number of embryos transferred were not statistically significant. Ten out of thirty-four patients in the TDT group had a positive outcome for pregnancy, corresponding to 29.4%. The Cook Sydney group had 9 patients out of 34 with positive outcomes, corresponding to 26.5%. Comparing the efficacy of both catheters for the primary outcome, there was no significant difference (*p*>0.05) between the TDT and the Cook Sydney catheters. The initial dose of gonadotropin reflects the ovarian reserve, and it was the only variable that showed statistical significance (*p*=0.040), although all patients were subjected to the same treatment regimen. The final dosage was not significant (*p*>0.05) in both groups. The primary outcome showed no difference between the groups. The statistical analysis results are depicted on Table I.

## DISCUSSION

The superiority of flexible catheters over rigid and semi-rigid ones is well established in the literature. ^Abou-Setta *et al.* (2005)^ in their review, concluded that flexible catheters have better pregnancy rates when compared to rigid catheters, and ^Ruhlmann *et al.* (2015)^ demonstrated that the implantation rate is increased when using flexible catheters rather than semi-rigid ones.

Several studies compared soft catheters. When the Cook Soft and the Edwards-Wallace catheters were compared, there was no statistically significant difference between them regarding pregnancy rates (^Boone *et al.*, 2001^). Another study comparing those two flexible catheters was carried out by ^McIlveen *et al.* (2005)^, with a larger sample (75 patients per group) and they also found no significant difference in pregnancy rates between the groups. ^Rhodes *et al.* in 2007^, comparing the same two types of catheters, showed that pregnancy rates with the Cook's was 5% higher than with the Wallace's: 63.3% vs. 58.0%, respectively; but the study power was not high enough to demonstrate statistically significant difference. ^van Weering *et al.* (2002)^ studied pregnancy rates comparing the TDT embryo transfer set and the Cook K-soft 5000 soft trans universal embryo transfer set, and found that pregnancy rates were significantly higher in the second group.

According to a literature review we carried out, the studies that compared the Cook and the Wallace soft catheters showed no statistical difference in efficacy between them (Boone *et al.*, 2001; ^McIlveen *et al.*, 2005^; ^Rhodes *et al.*, 2007^; ^Saldeen *et al.*, 2008^). Just one study reported on the TDT Set (Buckett, 2006) and we did not find studies that compared the two types of catheters we compared.

Our study compared two types of soft embryo transfer catheters, with pregnancy rates of 29.4% and 26.5%, for the TDT Set and the Cook Sydney, respectively. We found no significant difference between the groups (*p*>0.05). The groups were matched by age, they used the same ovarian stimulation protocol and underwent the same laboratory procedures. Although there was a statistically different initial dose of gonadotropin between the groups, it did not impact our primary outcome (pregnancy rate): subsequent laboratory data related to ovulation induction was similar for both groups. Thus, we had a homogeneous sample in hormonal and laboratory terms, which allowed us to assess whether there was a correlation between the catheter used for embryo transfer and the β-hCG outcome. We believe that the main weaknesses of this study were the sample size and the fact that it was not a randomized clinical trial (RCT).

## CONCLUSION

In conclusion, this prospective study supported that the modern ET soft catheters TDT and Cook Sydney Set had a similar performance in ART procedures. Catheter choice may be based on other factors, rather than its performance on outcomes. Future RCT studies concerning catheter types and their efficacy under ultrasound guided transfer are warranted.
